# Undiagnosed Dementia: A Scoping Review of Prevalence, Barriers, Impacts, Diagnostic Tools, and Intervention

**DOI:** 10.1192/bjo.2025.10145

**Published:** 2025-06-20

**Authors:** Mohammed Ibrahim, Khalid Mohamed

**Affiliations:** ^1^Betsi Cadwaladr University Health Board, Wales, United Kingdom; ^2^Faculty of Medicine, University of Bahri, Khartoum, Sudan

## Abstract

**Aims:** Dementia, including Alzheimer’s disease and other forms of cognitive impairment, often goes undiagnosed due to demographic, societal, and healthcare factors. Addressing these challenges is essential to enhance early diagnosis, improve patient outcomes, and reduce the societal and economic burden of dementia. This scoping review explores the prevalence, barriers, impacts, diagnostic tools, and interventions associated with undiagnosed dementia, identifying knowledge gaps and providing recommendations for research, clinical practice, and policy development.

**Methods:** Following PRISMA Extension for Scoping Reviews, an extensive search was done through PubMed, Scopus, Web of Science, Science Direct and CINAHL. The inclusion criteria in this study were studies published in English, on human subjects, and where undiagnosed dementia has been explicitly discussed within the context of the research questions. Studies were excluded if they related only to diagnosed dementia or other cognitive impairments without any implications for the undiagnosed part. From 235 studies, 119 duplicates were removed, and 25 studies met the inclusion criteria.

**Results:** The review identified five key themes: (1) Prevalence and Demographics of Undiagnosed Dementia: Prevalence rates varied widely across populations, influenced by factors such as age, gender, socioeconomic status, and ethnicity. Undiagnosed dementia was more common in rural areas and low-income settings, with older age and lower education as significant risk factors. (2) Barriers to Diagnosis and Recognition: Key barriers included societal stigma, cultural beliefs normalizing cognitive decline, limited healthcare access, and inconsistencies in diagnostic practices. (3) Impact on Patients and Caregivers: Undiagnosed dementia caused psychological, physical, and economic strain on patients and caregivers, exacerbated by delays in accessing treatment and support services. (4) Screening and Diagnostic Tools: Tools like MMSE, MoCA, and emerging technologies such as AI and electronic health records (EHRs) showed promise but faced challenges related to accuracy, cultural adaptation, and implementation. (5) Interventions and Recommendations: Proposed solutions included public health campaigns, standardized screening protocols, culturally sensitive tools, multidisciplinary care approaches, and policy reforms to improve early diagnosis and equitable access to dementia care.

**Conclusion:** This scoping review provides a comprehensive overview of the existing literature on undiagnosed dementia, highlighting that its prevalence varies across different populations. Interventions are needed to reduce diagnostic delays and improve early diagnosis, particularly among high-risk individuals in the community. Additionally, further research is required to develop and validate culturally sensitive diagnostic tools and screening protocols tailored to diverse populations.

